# Reconstruction of Foot and Ankle Defects: A Prospective Analysis of Functional and Aesthetic Outcomes

**DOI:** 10.7759/cureus.40946

**Published:** 2023-06-25

**Authors:** Michael Laitonjam, Manal M Khan, Deepak Krishna, Ved Prakash Rao Cheruvu, Reena Minz

**Affiliations:** 1 Plastic and Reconstructive Surgery, Shija Hospital and Research Institute, Imphal, IND; 2 Department of Burns and Plastic Surgery, All India Institute of Medical Sciences, Bhopal, Bhopal, IND; 3 Department of Burns and Plastic Surgery, All India Institute of Medical Sciences, Bhubaneswar, Bhubaneswar, IND

**Keywords:** american orthopedic foot and ankle society (aofas) ankle /hindfoot score, foot trauma, foot and ankle surgeries, foot and ankle reconstruction, foot and ankle defects

## Abstract

Introduction: Reconstruction of foot and ankle defects requires selecting an appropriate durable and aesthetically appealing option. From the different options, the procedure's choice depends on the defect's size, location, and donor area's availability. Patients' main goal is to have an acceptable biomechanical outcome.

Materials and methods: In this prospective study, we have included patients who had undergone reconstruction of the ankle and foot defects between January 2019 and June 2021. Patient demographics, location and size of the defect, different procedures, complications, sensory recovery, ankle hindfoot score, and satisfaction score were recorded.

Results: 50 patients with foot and ankle defects were enrolled in this study. All flaps survived except one free anterolateral thigh flap. Five locoregional flaps developed minor complications, and all skin grafts healed well. The Ankle Hindfoot Score outcome has no significant relation with the anatomical location of the defects and the reconstructive procedure. All patients reconstructed using random local flap and with free flap were satisfied with the aesthetic outcome.

Conclusions: Because of limited soft tissue, local flap availability is restricted to small defects. Satisfaction rates are high in local and free flaps and are best suited for reconstructing the weight-bearing part of the foot. Bulky flaps should be avoided over the dorsum and ankle region.

## Introduction

The foot and ankle region is responsible for upright positioning and ambulation. Insufficient soft tissue makes this area also prone to injuries and diseases [1].

Trauma, infection, tumor resection, compromise in vascular supply, sensation, and mechanical alignment of the ankle and foot are the factors that alter the stability of the skeletal and increase the development of soft-tissue damage or breakdown. Salvage is complex in such an injured or compromised foot often leads to significant amputation, which bears its physical, emotional, psychological, and socioeconomic morbidity, even resulting in lifetime dependence on prosthetic devices. Despite all the available resources and measures of rehabilitation, many patients never regain their ability or potential for ambulation and are condemned to a miserable life or existence bound to a wheelchair [2].

Leg ulcers, including ankle and foot ulcers, have plagued humanity since ancient times and still stand as a miserable burden to patients and caregivers in most parts of the world. Ulcers are wounds or raw areas with a “full-thickness defect” and a “slow tendency for healing.” Skin ulcers can lead to complete loss of the epidermis, parts of the dermis, and even subcutaneous fat or tissues [3].

Soft tissue reconstruction of the foot and ankle still poses a complex and challenging process to the plastic and reconstructive surgeon despite advances in transferring fasciocutaneous, musculocutaneous, and composite flaps [4]. Foot reconstruction aims to provide adequate soft tissue coverage and maintain functional recovery. The reconstructive plan should be individualized to ensure the best and ideal biomechanical outcome considering the patient’s age, goals, and operational capability. Depending upon the wound presentations, this may include wound dressing or local wound care, skin grafting, locoregional flaps, microsurgical free tissue transfer, or amputation. To select the suitable method of reconstruction, factors like defect size, location, donor site availability, involvement of surrounding structures, and morbidity of the surgical procedures should be considered for all the cases.

In this study, the goal or primary objective is to assess the outcome, like flap survival and immediate complication of various surgical modalities for the coverage of foot and ankle defects. Moreover, the study aimed to determine the functional outcome of pain, ulceration, sensation, daily activities, and cosmesis of various surgical modalities that cover foot and ankle defects.

## Materials and methods

We conducted a prospective study on 50 patients aged 12-60 years who presented with defects in the foot and ankle region and were admitted to the Department of Burns and Plastic Surgery, All India Institute of Medical Sciences, Bhopal, from January 2019 to Jun 2021. This study was approved by the Institutional Research Review Board (Ethics code: IHEC-LOP/2019/MCH002), and informed consent was obtained from all participants before enrolment.

Patients with defects of the foot and ankle region were examined and briefed about required surgical options for wound coverage after bilingual written informed consent was taken. All patients were operated on under appropriate anesthesia after debridement; defects were covered with the appropriate flap depending on the defect's size and location and the flap's availability. The small defects were managed with local flaps, medium to large defects with locoregional flaps, and large defects with free flaps when another option was unavailable. Superficial defects over the dorsum of the foot were covered with skin grafting. All patients were regularly followed up, and data recording was done at intervals of two weeks, one month, three months, and six months postoperatively. All the patients were followed up for a minimum of six months. The sensation was objectively assessed over the flap by using the Semmes-Weinstein monofilament test at the flap site, with three different monofilament sizes (size 6.65 ¼ 300 g for deep sensation, size 4.31 ¼ 2 g for protective sensation, and size 3.61 ¼ 0.4 g for normal sensation). The functional outcome regarding pain and ulceration were also assessed. The Ankle-Hindfoot Score (AHS), based on the American Orthopaedic Foot and Ankle Society (AOFAS), evaluated postoperative functional outcomes at six months. The cosmetic appearance of the reconstructed site was assessed by the patient or the patient’s relative.

Statistical analysis

Data were collected and entered in Microsoft Excel 2007 (Microsoft, Redmond, WA, USA) after checking for correctness and consistency. Data were analyzed using SPSS version 16 (SPSS Inc., Chicago, IL, USA). Data were described using mean, median, and percentages. A test of significance was performed using ANOVA as required. A probability value of less than 0.05 was taken as significant.

## Results

A total of 50 patients with foot and ankle defects were enrolled in this study. The male-to-female distribution was 41/9, and the overall median age of participants was 35 years, ranging from 12 to 60 yrs. The foot and ankle soft tissue defects were due to different etiologies, with trauma being the most common cause (28; 56%) and the dorsum of the foot being the most common site of the defect (18; 36%). The distribution of gender, age, etiology, location of the defect, and types of procedure are summarized in Table 1.

**Table 1:** Socio-demographic characteristics of participants.

Patient characteristics	Overall (N=50)
Age	
Mean (Range)	35 (12-60)
11 - 20	6 (12%)
21 - 30	16 (32%)
31 - 40	9 (18%)
41 - 50	12 (24%)
51 - 60	7 (14%)
Gender	
Male	41 (82%)
Female	9 (18%)
Etiology	
Trauma	28 (56 %)
Post infective	11 (22 %)
Neuropathic ulcer	8 (16 %)
Post-excision of the tumor	2 (4%)
Venous ulcer	1 (2 %)
Location of defect	
Dorsum	18 (36 %)
Heel	17 (34 %)
Plantar aspect forefoot	5 (10 %)
Lateral surface of the ankle	5 (10 %)
Medial surface of the ankle	4 (8 %)
Instep	1 (2 %)
Reconstructive procedure	
Pedicle flaps	24 (48 %)
Reverse sural artery flap	18 (36 %)
Perforator plus flap	2 (4 %)
Medial plantar artery flap	2 (4 %)
Reverse medial plantar artery flap	1 (2 %)
Cross leg flap	1 (2 %)
Local random flaps	6 (12 %)
V-Y Advancement flap	4 (8%)
Rotation flap	1 (2%)
Transposition flap	1 (2%)
Free flaps	6 (12 %)
Anterolateral thigh flap	3 (6 %)
Latissimus dorsi muscle flap	2 (4 %)
Radial artery forearm flap	1 (2 %)
Split-thickness skin graft	14 (28 %)

Complications

In immediate complications, out of 30 cases of the pedicle and local random flaps, four patients (three reverse sural artery [RSA] and one V-Y advancement) developed wound dehiscence in the distal margins of the flaps due to infection. Regular cleaning of flap margins was done along with local and intravenous antibiotic coverage, and the wound dehiscence was healed by secondary intention. There was one reverse sural artery flap in which distal necrosis was developed of around 2 cm. Debridement of necrotic tissues was done after complete demarcation, and the resultant defect was allowed to granulate for a few days and covered with skin grafting later. There was no graft failure in 14 cases; those resurfaced with split-thickness skin grafting. On the contrary, out of six free flaps, one anterolateral thigh flap was lost due to venous thrombosis, despite an attempt of re-exploration and leech therapy to relieve venous congestion. The necrosed flap was removed on the fourth postoperative day, and split-thickness skin graft (SSG) was done later on, once the wound became granulating with the help of vacuum-assisted closure (VAC) therapy.

Sensation

Assessment of various sensation outcomes such as deep sensation, protective sensation, and pain sensation was done in two weeks, one month, three months, and six months intervals postoperatively. As one anterolateral thigh flap was lost entirely, sensation assessment was done in 49 patients only (Table 2).

**Table 2:** Outcome of the deep, protective, and pain sensation in different groups. SSG: Split-thickness skin graft

Procedure Type, No.	Sensation type	Regained sensation in Follow up time				No regain of sensation N, (%)
		2 weeks N, (%)	1 month N, (%)	3 months N, (%)	6 months N, (%)	
Pedicle flap (24)	Deep	0	12(50.0)	19(79.2)	21(87.5)	3(12.5)
	Protective	0	0	10(41.7)	15(62.5)	9(37.5)
	Pain	0	0	0	1(4.2)	23(95.8)
Local random flap (6)	Deep	0	2(33.3)	4(66.7)	4(66.7)	2(33.3)
	Protective	0	0	1(16.7)	4(66.7)	2(33.3)
	Pain	0	0	0	0	6(100.0)
Free flap (5)	Deep	0	0	1(20.0)	2(40.0)	2(40.0)
	Protective	0	0	0	1(20.0)	4(80.0)
	Pain	0	0	0	0	5(100.0)
SSG (14)	Deep	7(50.0)	9(64.3)	13(92.8)	14(100.0)	0
	Protective	0	1(7.1)	7(50.0)	11(78.6)	3(21.4)
	Pain	0	0	3(21.4)	8(57.1)	6(42.9)

Deep sensation

**Figure 1:**
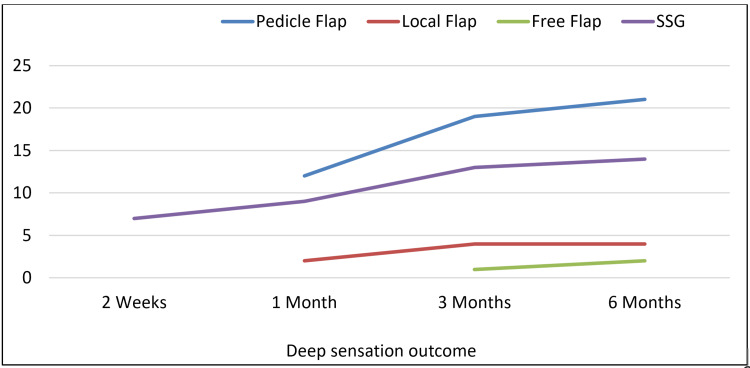
Comparison of the deep sensation outcome in different groups SSG: Split-thickness skin graft

In the deep sensation assessment, out of 24 cases of pedicle flaps, 12 (50%) regained deep sensation at one month, which increased to 19 (79.2%) and 21 (87.5%) at three months and six months, respectively. Outside of six cases of the random local flap, two (33.3%) cases regained deep sensation at one month, which increased to four (66.7%) cases at three months and remained the same at six months follow-up. Out of five cases of survived free flap, one (20%) patient developed deep sensation at three months, which increased to two (40%) cases at six months. And among the 14 SSG cases, seven (50%) patients regained deep sensation as early as two weeks which later increased to nine (64.3%) and 13 (92.8%) patients in one month and three months, respectively. By six months postoperative period, all 14 (100%) patients who underwent SSG regained deep sensation (Figure 1).

Protective sensation

**Figure 2:**
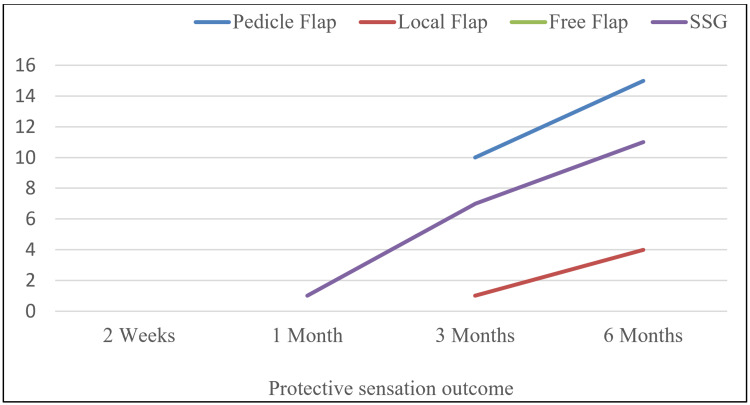
Comparison of protective sensation outcome in different groups SSG: Split-thickness skin graft

In the protective sensation assessment, 10 (41.7%) pedicle flaps regained protective sensation by three months which increased to 15 (62.5%) by six months. Among the random local flap, one (16.7%) case regained protective sensation at three months and increased to four (66.7%) cases at six months. Only one (20%) free flap regained protective sensation at six months. Among the 14 cases of SSG, one (7.1%) case regained protective sensation at a one-month post-operative period which increased to seven (50%) and 11 (78.6%) at three months and six months, respectively (Figure 2). 

Pain sensation

**Figure 3:**
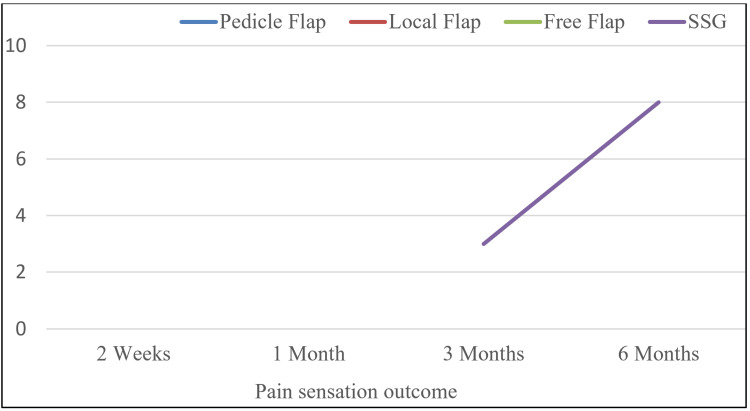
Comparison of the pain sensation outcome in different groups SSG: Split-thickness skin graft

In pain sensation assessment, defects reconstructed using local random flaps and free flaps developed no pain sensation even after six months of follow-up. On the contrary, only one (4.2%) patient reconstructed with pedicle flap regained pain sensation at six months post-operative follow-up. Out of the 14 patients reconstructed using SSG, three (21.4%) cases regained pain sensation at three months, which increased to eight (57.1%) at six months (Figure 3).

Ankle-Hindfoot Score

Regarding the AHS among the various modalities of foot and ankle defects reconstruction in our study, the score was highest in patients reconstructed using SSG with a mean score of 89.80 ± 8.5, followed by patients reconstructed with pedicle flap (88.75 ± 6.7) and random local flap (88.17 ± 8.1) and lowest in patients reconstructed using a free flap with a mean score of 83.20 ± 4.8 (Table 3). The AHS outcome has no significant relation with the anatomical location of the defects and different options for reconstruction of defects (Table 3).

**Table 3:** AHS in relation to the site of defect and type of reconstruction. AHS: Ankle-Hindfoot Score, SD: Standard deviation, SSG: Split thickness skin graft. The mean difference is significant at the 0.05 level

Site of defect	Frequency	AHS (max score 100) Mean ± SD	ANOVA F
Dorsum of foot	18	89.17 ± 8.2	F=0.734 P=0.602
Heel	17	87.94 ± 6.2	
Planter aspect forefoot	5	86.40 ± 6.3	
Lateral surface of the ankle	5	85.20 ± 7.2	
Medial surface of the ankle	4	91.75 ± 10.2	
Instep region	1	NA	
Type of reconstruction			
Pedicle flap	24	88.75 ± 6.7	F=0.698 P=0.574
Local random flap	6	88.17 ± 8.1	
Free flap	6	83.20 ± 4.8	
SSG	14	89.80 ± 8.5	

Satisfaction score

Considering the satisfaction score, based on the aesthetic outcomes, it was assessed in 49 patients only as one free flap was completely lost, and therefore, this patient was not included in the assessment. All six (100%) patients reconstructed using local random flap and five (100%) patients with free flap were satisfied with the result. Among the pedicle flap, 18 (75%) were satisfied with the result. At the same time, three patients (12.5%) with pedicle flap and one patient (7.1%) with split-thickness graft were very satisfied with their final outcomes. Three (12.5%) patients with pedicle flap and one (7.1%) patient with a split-thickness skin graft, respectively, were not satisfied with the final aesthetic outcome (Table 4). No statistical difference was found in Table 2 and Table 4. 

**Table 4:** Comparison of satisfaction score in different groups. SSG: Split thickness skin graft

Type of Modality	Not satisfied (%)	Satisfied (%)	Very satisfied (%)
Flaps (35)			
Pedicle flap (24)	3 (12.5%)	18 (75%)	3 (12.5%)
Local flap (6)	0 (0.0%)	6 (100.0%)	0 (0.0%)
Free flap (5)	0 (0.0%)	5 (100.0%)	0 (0.0%)
SSG (14)	1 (7.1%)	12 (85.7%)	1 (7.1%)

## Discussion

Injuries over the foot and ankle region still pose a common problem, usually in complex soft tissue defects. Soft tissue defects of the foot and ankle present as an enigma to Plastic and Reconstructive surgeons. Leaving such injuries unattended may lead to grave conditions affecting the lifestyle, such as chronic pain, non-healing ulcers, and compromised function. The primary goal of any reconstruction of the foot and ankle region is to cover the wound and achieve bipedal ambulation. Depending on the site, size, depth, and defect condition, various reconstructive modalities are available for such defects. Sound knowledge of the reconstructive ladder and its modification and the surgeon’s skill and experience helped in the planning and decision-making.

In our study, the dorsum of the foot constituted the most common anatomical location of the defect, followed by the heel and the defect around the ankle. Likewise, in a study conducted by Hamad et al. [5] and Zhu et al. [6], they also found the dorsum of the foot to be the most common location of the defect, followed by the malleoli region and heel. Regarding etiology, in our study, trauma (56%) was the most common cause of foot and ankle defects. Similar studies conducted by Bhandari et al. [7] and Dogra et al. [8] showed that trauma was the primary cause of foot and ankle defects, comprising 56.6% and 50%, respectively. Also, in a study by Moon and Pandey [9], trauma was the most common cause of foot and ankle defects.

**Figure 4:**
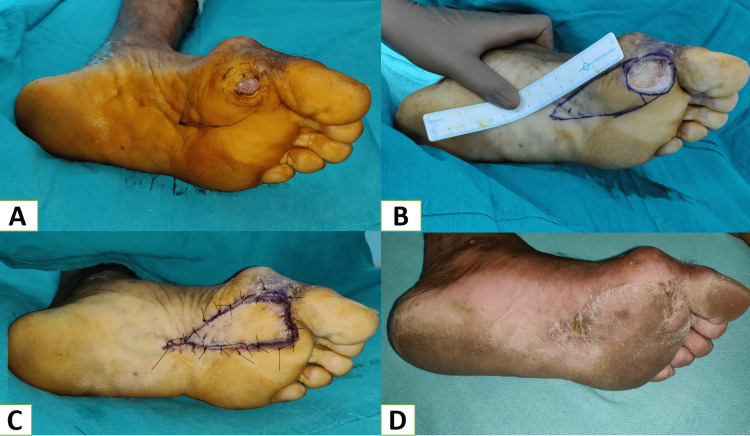
A: Diabetic ulcer at first MTP joint, B: Marking for V-Y advancement flap after debridement of the ulcer, C: After insetting the flap, D: Six months follow-up picture. MTP: Metatarsophalangeal

Local random pattern flaps are ideally helpful in reconstructing small defects using the adjacent tissue with similar features - replacing “like with like.” But due to the tightness of glabrous skin over the plantar aspect of the foot, the usage of local flaps in the foot has certain limitations. In our study, only 12% of cases with small-size defects over the plantar region were reconstructed with local flaps. Among the local flaps, V-Y advancement was the most common (Figure 4A-4D). In a similar study by Patkar, V-Y flaps (7.7%) were used for coverage of small defects (around 2 cm) over the mid-foot and fore-foot region [10].

**Figure 5:**
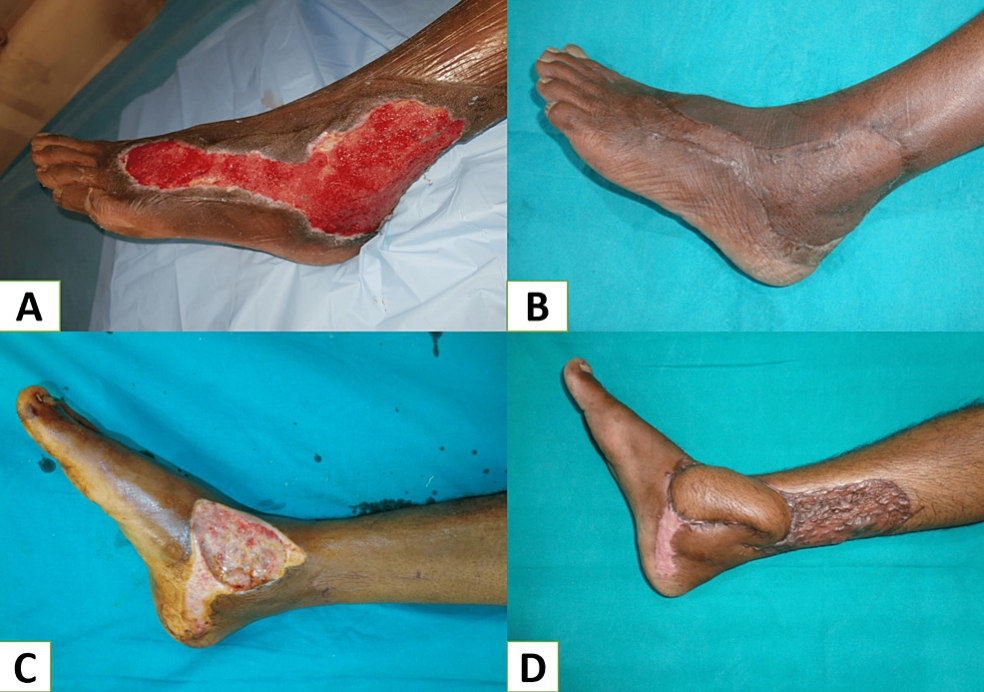
A: Post infective raw area dorsolateral aspect of left foot, B: Healed skin graft (six months follow-up), C: Post-traumatic soft tissue defect over right malleolar region, D: Perforator plus flap based on posterior tibial artery (three months follow-up).

SSG was used in resurfacing superficial foot and ankle defects with a success rate of 100% in our study. Maximum grafting cases were done for defects over the dorsum and dorsolateral aspect of the foot (Figure 5A, 5B). In a similar survey conducted by Bhandari et al. [7], they used SSG to resurface superficial defects over the dorsum of the foot in 10% of cases in their study group. In another study by Hamad et al. [5], they used SSG for coverage in 42.6% of their patients, and the defects were mainly located over the dorsum of the foot without exposure to any tendon, bone, or neurovascular structures. 

Almost half of the cases in our study were reconstructed using pedicle fasciocutaneous flaps. Pedicle flaps were often used for medium to large-sized foot and ankle defects (Figure 5C, 5D). The merit of pedicle flaps is easy dissection and harvesting of the flap, no need for sacrificing any major artery, minimal operating time, comparatively better success rates, good color match, and minimal requirement for aggressive monitoring. But there are certain demerits, such as a higher rate of developing venous congestion, two-stage procedure, limitation of reach, and donor site morbidity.

**Figure 6:**
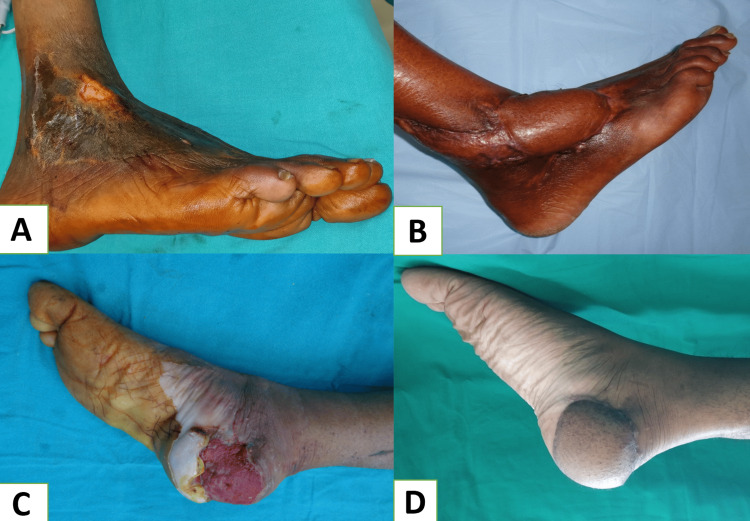
A: Post-traumatic unstable scar with a non-healing ulcer over the dorsum of the right foot, B: Islanded reverse sural artery flap for defect over the dorsum of the right foot, C: Post-traumatic heel defect, D: The defect was reconstructed with extended RSA flap. RSA: Reverse sural artery.

In this study, among the pedicle flaps, distally based superficial sural artery (RSA) flaps were used in maximum cases (Figure 6A-6D). It is a versatile flap, and it is ideal for the coverage of defects of the heel pad, dorsum of the foot, the anterolateral aspect of the ankle, and the lateral aspect of the hind foot. This flap has certain advantages in being easy to dissect and harvest and having minimal donor site morbidity. At the same time, this flap has certain complications, and venous congestion is the most important. In our series, one peninsular RSA flap developed partial necrosis, and two peninsular and one islanded RSA flap developed wound dehiscence in the distal margin. In a more or less similar study, Turan et al. [11] also reported the development of partial necrosis in three RSA flaps out of 25 cases due to venous congestion. Moreover, in a study conducted by Ramesha et al. [12], they experienced venous congestion of the RSA flap with distal flap necrosis in 25% of cases compared to 5.6% in our study. Extended/two-stage pedicled reverse sural flap reduces the risk of venous congestion, increases the flap's reach, and maintains the contour of the posterior heel when used for the weight-bearing part of the heel [13].

**Figure 7:**
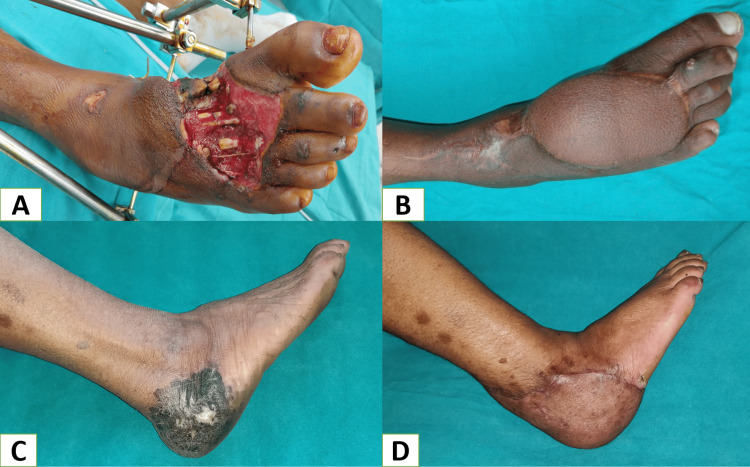
A: Post-traumatic composite defect dorsum of the right foot, B: Heals wound after coverage with free anterolateral thigh musculocutaneous flap, C: Melanoma over the right heel, D: Heel reconstructed with free latissimus dorsi muscle flap with skin grafting over it.

Our study used free flaps to reconstruct large, extensive foot and ankle defects or regions where other pedicle flap options were unavailable (Figure 7A-7D). Free flaps have certain advantages regarding good contour matching and minimal donor site morbidity and can be used as composite flaps, and two team approach is possible. Disadvantages include prolonged operating time, aggressive monitoring, microsurgical skill and expertise, and sacrifice of major vessels.

The instep region of the foot is the non-weight-bearing part of the plantar surface of the foot from where the medial plantar artery flap is raised. It is a fasciocutaneous island flap. The medial plantar artery and venae comitantes formed this flap's principal dominant vascular pedicle [14]. This flap has been ideally used to cover soft tissue defects over the plantar aspect of the foot, forefoot, ankle, and weight-bearing area of the heel. This flap is usually a proximally or distally based pedicled island flap. In our study, two cases of heel defect were reconstructed using a proximally based medial plantar artery flap and one case of the plantar aspect of forefoot defect with a distally based medial plantar artery flap.

Cross-leg flap is the last option in foot and ankle reconstruction in demanding conditions. This flap has certain demerits, such as two stages of the procedure, patient morbidity, and discomfort due to immobilization of the legs, and its use is limited in older patients due to the high chance of developing joint stiffness and even deep vein thrombosis [5]. One case of soft tissue defect over the medial aspect ankle was reconstructed with a superior-based fasciocutaneous cross-leg flap in our study.

In our study, the deep sensation was regained in all the patients who underwent SSG and, in most cases, underwent reconstruction with pedicle fasciocutaneous flap and local random flaps by six months follow-up. But on the contrary, it was less in free flap cases. This might be because of extensive traumatic injury where we used free flaps for reconstruction. Regarding protective sensation, around two-thirds of the cases in both pedicle and local random flaps, and three-fourths of patients who underwent SSG regained protective sensation by six months postoperative period. On the other hand, only one free flap regained protective sensation at six months follow-up. Likewise, in a more or less similar study conducted by Patkar [10], pressure sensation was regained in all patients at three months post-operative follow-up. Depending on the size, smaller flaps had developed some degree of sensation at six months and had regained good protective sensation at one year. There is no significant difference between those flaps which underwent neurotization and those without in terms of return of sensation [10]. Likewise, in another study by Zhu et al. [6], all patients regained protective sensation by three to 12 months postoperative period.

In our series, we assessed the patient's functional outcome at six months using AHS clinical ratings. We calculated the mean AHS for various types of flap and skin grafts and analyzed its relationship with the location of defects. Likewise, a study by Luen et al. [15] assessed pain and functional outcomes using questionnaires based on the subjective components of the AOFAS hindfoot scale. They analyzed the mean AOFAS score based on different types of flaps used to reconstruct the heel pad. In their study, the mean AOFAS score for distally based reverse sural artery flap was 45 (out of 60 points) compared to 55.8 (out of 60 points) in our study. Again, a study performed by Jandali et al. [16] where reconstructed foot and ankle defects using medial sural artery perforator-free flaps. The final objective outcomes of all patients were evaluated at 12 months post-operative with the AOFAS clinical rating system. In his study, patients achieved a mean AHS of 80.0 ± 6.7 points compared to the mean AHS of 83.2 ± 4.8 points among the free flaps group in our study. Moreover, our study's mean AHS score in patients reconstructed with SSG was 89.80 ± 8.5 points. Wounds resurfaced using SSG had no complications like fracture, exposed bone, or tendon and were used at non-weight-bearing parts. More or less, they were superficial wounds with healthy granulation tissues. So, this might cause the high mean AHS in patients reconstructed with SSG compared to other flaps in our study.

Turan et al. [11] performed reconstruction of foot and ankle defects in 25 patients with RSA flap and assessed the satisfaction rating of the patient as not satisfied, poor, moderate, good, excellent, and very satisfied. All the patients were categorized as excellent in aesthetic appearance. Likewise, our study assessed patient satisfaction ratings as not satisfied, satisfied, and very satisfied. Of 18 patients with RSA flap, 72.2% were satisfied with the aesthetic outcomes. And regarding free flaps, in our study, 100% of patients who underwent reconstruction with free flaps were satisfied with the aesthetic result as compared to 72% in a study done by Elgohary et al. [17] on the reconstruction of soft tissue defects of the heel with free flaps. One anterolateral free flap was completely lost; therefore, it was excluded from the satisfaction score assessment.

Limitations of our study

The sample size was small, so a significant comparison between different options was impossible. Further study with a large sample size will be required to test substantial comparisons. Follow-up of patients was done for six months; hence long-term durability of skin graft/flap could not be assessed, and even complications such as ulceration or recurrence.

## Conclusions

The aesthetic outcome was more with local and free flaps than pedicle flaps and skin grafting. Although with the advance of microsurgical technique the quality of foot and ankle defect reconstruction has improved a lot, pedicle and local flaps still give comparable results.

Besides the surgical procedure, management should include lifestyle modification, custom-made protective shoes to prevent ulceration, and long-term regular follow-up from assessing for complications such as ulceration or recurrences and their time management.
